# Downregulation of secreted frizzled-related protein 4 inhibits hypoxia/reoxygenation injury in diabetic cardiomyocytes by protein tyrosine phosphatase nonreceptor type 12

**DOI:** 10.1080/21655979.2022.2034706

**Published:** 2022-03-15

**Authors:** Zhifeng Bai, Xiuhong Hao

**Affiliations:** Second Department of Cardiovascular Medicine, The First People’s Hospital of Shangqiu City, Shangqiu, China

**Keywords:** SFRP4, diabetic cardiomyocytes, PI3K/AKT, PTPN12, H/R injury

## Abstract

Myocardial ischemia-reperfusion injury in diabetic patients leads to an increased incidence of complications and mortality. Secreted frizzled-related protein 4 (SFRP4) plays a critical role in diabetic myocardial ischemia-reperfusion. This paper aims to uncover the underlying mechanisms of SFRP4 in hypoxia/reoxygenation (H/R) injury of diabetic myocardial cells. An in vitro ischemia/reperfusion (I/R) injury model was established using high glucose-induced H9c2 cardiomyocytes. Expression of SFRP4 was detected by real-time reverse transcriptase-polymerase chain reaction and Western blotting. After transfection of SFRP4, the binding of SFRP4 to protein tyrosine phosphatase nonreceptor type 12 (PTPN12) was predicted by database and verified by co-immunoprecipitation assay. P13 K/AKT protein levels were examined by Western blotting. PTPN12 levels were tested by RT-qPCR and Western blotting, cell viability by Cell Counting Kit-8, lactose dehydrogenase kit, terminal dUTP nick-end labeling assay, and cell inflammation and oxidative stress by Western blotting and enzyme linked immunosorbent assay. After overexpression of PTPN12, the experiments for cell viability, inflammation and oxidative stress were repeated once more. SFRP4 expression was upregulated in a high-glucose-stimulated H/R cardiomyocyte model. The interference of SFRP4 promoted cell viability, inhibited the inflammatory and oxidative stress response of H/R cardiomyocytes induced by high glucose. SFRP4 interacted with PTPN12 and inhibited the PI3K/AKT signaling pathway. PTPN12 overexpression reversed the inhibitory effect of sh-SFRP4 on H/R cardiomyocyte damage induced by high glucose. Downregulation of SFRP4 inhibited H/R cell damage in diabetic cardiomyocytes by binding to PTPN12.

## Graphical abstract

**Figure:**
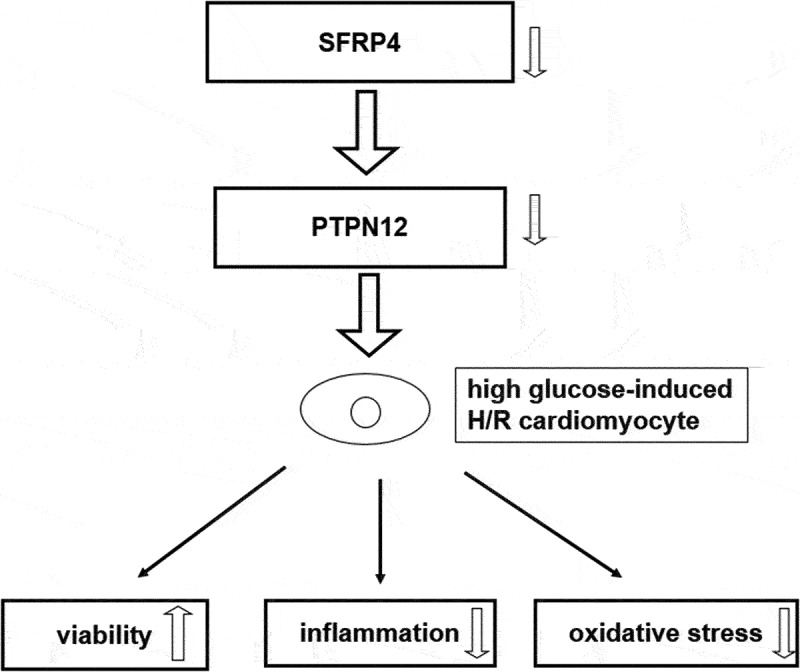

## Introduction

The incidence of diabetes is increasing year by year. Cardiovascular diseases, mainly ischemic heart disease, are the leading cause of morbidity and mortality in diabetics [1]. Diabetic cardiomyopathy is a heart dysfunction that occurs in the absence of hypertensive heart disease, coronary artery disease and valvular heart disease [2], is also a diabetic-induced pathophysiological condition that can lead to heart failure. New evidence suggests that oxidative stress, inflammatory response, autophagy, apoptosis, and abnormal myocardial metabolism are involved in the development of diabetic cardiomyopathy [3]. Additionally, hyperglycemia can adversely affect myocardial tissue through a variety of mechanisms [4,5]. Diabetic cardiomyopathy is mainly treated by controlling blood glucose, lowering blood pressure, lowering blood lipid and anti-heart failure. However, the prognosis is poor. As a result, new strategies to protect diabetic hearts from increased myocardial ischemia/reperfusion injury are urgently needed.

Secreted frizzled-related protein 4 (SFRP4) is a protein coding gene belonging to the SFRP family. It binds extracellularly to Wnt ligands or Frizzled receptors, thereby regulating the Wnt cascade [6]. There is a growing body of literature that recognizes the importance of SFRP4 in diabetes. For example, SFRP4 expression is up-regulated in diabetic patients [7] and in plasma from patients with coronary artery disease [8]. On the basis of bioinformatics analysis, SFRP4 is also up-regulated in diabetic cardiomyopathy [9]. Additionally, downregulation of SFRP4 improves cardiac function after ischemic injury [10]. Meanwhile, it has been observed that knockdown of SFRP4 in mice protected myocardium from ischemia-reperfusion injury [11]. Another research stated that SFRP4 can elevate ROS levels to cause endothelial dysfunction in vascular endothelial cells [12]. These findings suggest that SFRP4 may be involved in diabetic myocardial ischemia-reperfusion injury. To identify the specific mechanism of SFRP4 in diabetic cardiomyopathy, the analysis of the MINT database (https://mint.bio.uniroma2.it/) and the BioGRID database (https://thebiogrid.org/) revealed its ability to bind to protein tyrosine phosphatase nonreceptor type 12 (PTPN12).

PTPN12 is a newly identified molecule involved in tumorigenesis and development [13]. Extensive research has shown that PTPN12 is also implicated in embryonic development, apoptosis, cell cycle and metabolism [14]. And data from one study suggested that PTPN12 expression was upregulated after the myocardial ischemia/reperfusion (I/R) injury and exerted a role in promoting cell death under H/R stress [15]. Recent study noted that PTPN12 activated the PI3K/AKT signaling pathway after silencing [16]. There are numerous studies showing that activation of PI3K/AKT can reduce injury induced by I/R and diabetic cardiomyopathy injury [17]. According to the GEPIA database (http://gepia.cancer-pku.cn/), the expressions of SFRP4 and PTPN12 were found to be positively correlated in the heart.

Therefore, in this study, we aimed to identify the role and mechanism of SFRP4 in diabetic cardiomyopathy. We made the conjecture that downregulation of SFRP4 inhibited diabetic cardiomyocyte H/R injury through activation of the PI3K/AKT signaling pathway by PTPN12.

## Materials and methods

### Cell culture and H/R model treatment

The rat embryonic ventricular cardiomyocytes H9c2 cell line was obtained from Sigma-Aldrich (St.Louis, MO, USA) and kept in Dulbecco’s modified Eagle’s medium (DMEM) (Gibco Laboratories, USA) containing 10% fetal bovine serum (FBS) (Thermo Fisher Scientific, Waltham, MA, USA), 100 U/mL penicillin and 100 lg/mL streptomycin at 37°C in 5% CO_2_.

H9c2 cells were placed in six-well plates up to 50%-60% confluence. Then, these cells were cultured in a serum-free medium overnight before treatment and stimulated with high glucose at a concentration of 30 mmol/L for 24 h. Afterward, cells were subjected to an incubator with a mixture of 95% N_2_, 5% CO_2_ and 1% O_2_ at 37°C for 12 h to simulate anoxia. Finally, cells were exposed in a normoxic chamber for 12 h reoxygenation [18].

### Cell transfection

Short hairpin RNAs (shRNAs) against SFRP4 namely sh-SFRP4-1 (5′-GGAAGTTAGTGATATATAA-3′) and sh-SFRP4-2 (5′-GCAGAAGAATGATACATAA-3′), sh-NC (5′-GGTACGCAATAGGAGTGTGTG-3′), as well as PTPN12 overexpression vector and overexpression negative control were all purchased from Gene Pharma (Shanghai, China). Then, H9c2 cells were seeded into 6-well plates at a density of 4 × 10^5^/well and grown to 70–80% confluence. The above shRNAs were transfected into H9c2 cells using 2.5 μl/ml Lipofectamine 2000 reagent (Invitrogen, CA, USA) according to the manufacturer’s instructions.

### Real-time reverse transcriptase-polymerase chain reaction (RT-qPCR) analysis

The extracted total RNA of H9c2 was carried out following the standard procedures of vendor’s instructions by TRIzol® reagent (Invitrogen, CA, USA). A NanoDrop 3000 spectrophotometer (ThermoScientific, Waltham, MA) was used to confirm the sufficient quality of total RNA according to the manufacturer’s protocol. PrimeScript RT Master Mix (Takara,Tokyo, Japan) was used to reverse transcribe 2 μg of RNA into cDNA. Quantitative real-time PCR analysis was implemented to determine the mRNA expression using SYBR Premix Ex Taq kit (Unibiotest, Wuhan, China) in line with the instructions of manufacturer. The amplification conditions for this reaction were as follows: 95°C for 10 minutes, followed by 40 cycles of 95°C for 10 seconds and 60°C for 60 seconds. The primer sequences for PCR are presented as below: SFRP4, 5′-CACCTATCCCTCGAACGCAA-3′ (forward) and 5′-TCCTGTAAGGGTGGCTTCAAC-3′ (reverse); PTPN12, 5′-CTGCCATTTGATCACAGCCG-3′ (forward) and 5′-TGCTCTCGGCCCATATACAC-3′ (reverse); 5′-CACCTATCCCTCGAACGCAA-3′ (forward) and 5′-TCCTGTAAGGGTGGCTTCAAC −3′ (reverse); GAPDH, 5′-GTCGTGGAGTCTACTGGCGTCTTCA-3′ (forward) and 5′-TCGTGGTTCACACCCATCACAAACA-3′ (reverse). The relative expression of genes was normalized to the expression of GAPDH (internal control) be means of 2^−ΔΔCT^ method [19]. The experiment was repeated three times.

### Western blot analysis

The proteins extraction was prepared from H9c2 cells [20]. Then, proteins were lysed in the RIPA lysis buffer (Elabscience, Wuhan, China) on ice. The quantitation of proteins concentration was conducted with the aid of the BCA Protein Assay kit (Beyotime, Shanghai, China) in the light of the guidance of manufacturer. Subsequently, the protein samples were separated by SDS-PAGE gels, followed by the shifts onto polyvinylidene difluoride membranes (Millipore, Billerica, MA, USA). After washing with PBS and blocking with 5% skimmed milk, primary antibodies (SFRP4, 1:1000, ab154167; IL-1β, 1:1000, ab254360; TNF-α, 1:1000, ab215188; PTPN12, 1:1000, ab289859; PI3K, 1:1000, ab191606; AKT, 1:1000, ab179463; p-PI3K, 1:1000, ab182651; p-AKT, 1:1000, ab192623; GAPDH, 1:1000, ab8245) were put in the incubator and cultivated with these membranes overnight at room temperature, followed by an incubation of secondary antibodies labeled by horseradish peroxidase at indoor temperature for 1 h. The detection of protein bands was implemented through Image J software (National Institutes of Health, Bethesda, MD, USA).

### Determination of cell viability

The 96-well plates were used to load H9c2 cells with a density of 1 × 104 cells/well. The plates were placed in an incubator at 37°C for 2 h after the addition of 10 μl CCK-8 solution (Dojindo Molecular Technologies, Gaithersburg, MD, USA). The measurement of the absorbance was carried out at 450 nm with a microplate reader (BioTek, Biotek Winooski, Vermont, USA) [21].

### Lactate dehydrogenase (LDH) kit assay

LDH levels in the supernatant of H9c2 cells were examined with an LDH assay kit (Nanjing Jiancheng Institute of Biological Engineering) with reference to the manufacturer’s instructions [22]. Subsequently, the absorbance was measured with a microplate reader (Molecular Devices, San Jose, CA, USA).

### Cell apoptosis detection by TUNEL assay

H9c2 cells were first fixed with 1% formaldehyde for 1 h at 37°C in the dark and subsequently rinsed with PBS for three times. These cells were then mixed with 0.2% Triton X-100. The assay of cell apoptosis was performed with terminal deoxynucleotidyl transferase-mediated dUTP nick end labeling (TUNEL) (Abcam, Cambridge, MA, USA). The cells were observed with the aid of a fluorescence microscope (Sunnyoptical, Yuyao, China) [23].

### Enzyme-linked immunosorbent assay

The content of MDA, SOD and GSH in H9c2 cells were tested by means of corresponding commercial kits (Nanjing Jiancheng Bioengineering Institute, Nanjing, China) in accordance with the directions of manufacturer. Briefly, RIPA lysis buffer was applied to lyse these H9c2 cells and then the supernatant was taken. The levels of MDA, SOD and GSH were determined using a microplate reader (Bio-Rad, Hercules, CA, USA) by measuring absorbance at 532, 550 and 412 nm, respectively.

### Immunoprecipitation assay

H9c2 cells were collected in lysis buffer and placed on ice for 30 min. Then, these treated cells were centrifuged at 15, 000 rpm for 15 min at 4°C. The supernatant was gathered. Immunoprecipitation experiments were performed using 5 μg antibody and 500 μg protein. The collection, washing and elution of precipitated proteins were carried out. Finally, Western blotting was adopted to treat these protein blots [24].

### Statistical analysis

The experimental data are analyzed by SPSS Vision 19.0 (SPSS, Chicago, IL, USA) and mainly presented in the means mean ± SD. Differences among multiple groups were analyzed using one-way ANOVA. When P was less than 0.05, it was regarded as statistically significant difference.

## Results

In this study, we explored the biological functions of SFRP4 and the potential mechanism in high-glucose-stimulated H/R cardiomyocyte model. The data showed that the silencing of SFRP4 promoted the viability and suppressed high glucose-induced inflammation and oxidative stress under H/R condition. In addition, SFRP4 was found to be bound with PTPN12 and inhibit PI3K/AKT signaling pathway. Moreover, PTPN12 overexpression reversed the effects of SFRP4 silencing on high-glucose-stimulated H/R cardiomyocyte injury.

### SFRP4 expression was upregulated in a high-glucose-stimulated H/R cardiomyocyte model

**Figure 1.**
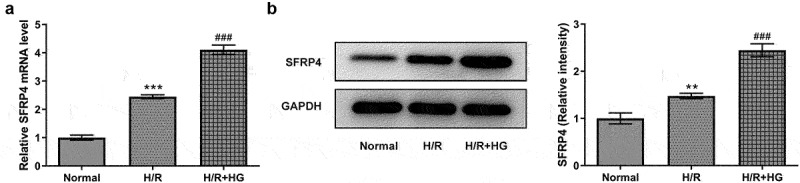
SFRP4 expression is upregulated in a high-glucose-stimulated H/R cardiomyocyte model. (a-b). Detection of SFRP4 expression was performed using RT-qPCR and Western blotting. Results are the mean ± SD. **P < 0.01, ***P < 0.001 versus Normal. ^###^P < 0.001 versus H/R.

H/R model in H9c2 cells was established under HG conditions. The experiment was divided into three groups: normal, HR, and HR+HG. Subsequently, SFRP4 expression in the cells was assayed by RT-qPCR and Western blotting. Figure 1a,b showed the same trend that the mRNA expression and protein level of SFRP4 were lowest in the normal group, elevated in the H/R group, and reached the highest after the addition of HG. This result suggested that SFRP4 expression was upregulated in the high glucose-stimulated H/R cardiomyocyte model.

### Interference of SFRP4 promoted the viability of high glucose-stimulated H/R cardiomyocytes H9c2 cells

**Figure 2.**
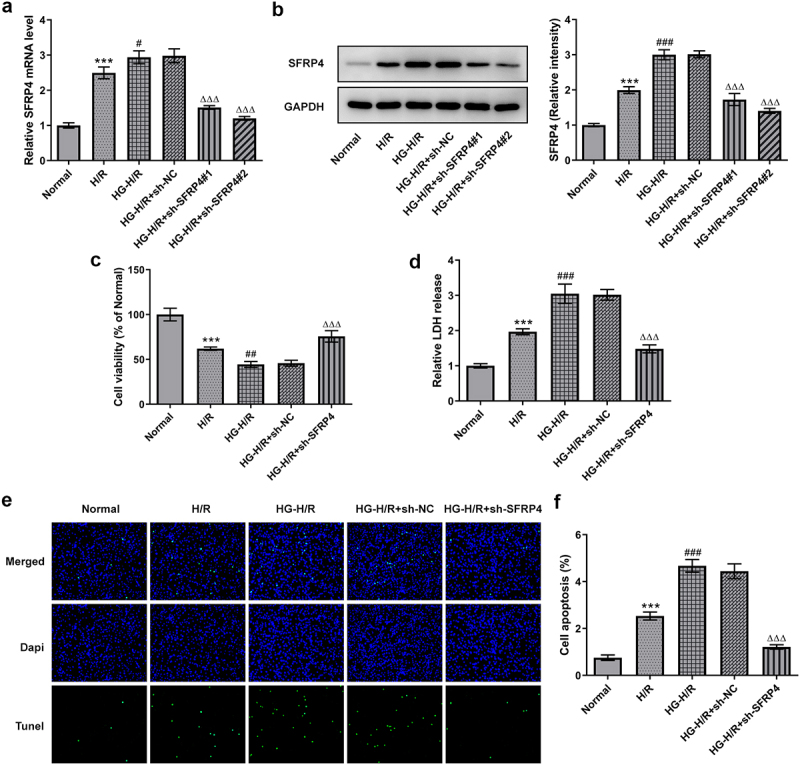
Interference with SFRP4 promotes cell viability of high glucose-stimulated H/R cardiomyocytes cells. (a). Detection of cell interference efficiency after transfection was conducted using RT-qPCR and Western blotting. (b). Cell viability was tested by means of CCK-8 kit. (c). The LDH kit was adopted to detect the release of LDH. (d). Determination of apoptosis levels was implemented with the help of TUNEL. Results are the mean ± SD. ***P < 0.001 versus Normal. ^#^P < 0.05, ^##^P < 0.01, ^###^P < 0.001 versus H/R. ^ΔΔΔ^P < 0.001 versus HG-H/R + sh-NC.

Cell viability at pre-transfection and post-transfection in H9c2 cells was determined by RT-qPCR, Western blotting, CCK-8, LDH kit and TUNEL. As shown in Figure 2a, both mRNA and protein level of SFRP9 were significantly diminished in the H/R cell model under high glucose stimulation after SFRP4 interference, compared with the NC group. Among them, sh-SFRP4-2 was selected for the next experiments because of its higher interference efficiency. In addition, it was apparent from Figure 2b that the viability of high-glucose-induced H9c2 cells in H/R model was decreased sharply, but recovered slightly after SFRP4 interference. In the contrary, from Figure 2c, it can be seen that LDH release was all gradually increasing, but dropped rapidly after sh-SFPR4 interference. Not only that, what can be clearly seen in Figure 2d was a steep fall in the level of apoptosis of high glucose induced H9c2 cells in H/R model after interference of SFRP4, in comparison with the negative control. Overall, these results indicated that interfering with SFRP4 did have a more potent effect on promoting the viability of high glucose stimulated H/R cardiomyocytes H9c2 cells.

### Interference with SFRP4 inhibits inflammation and oxidative stress in high glucose-induced H/R cardiomyocytes

**Figure 3.**
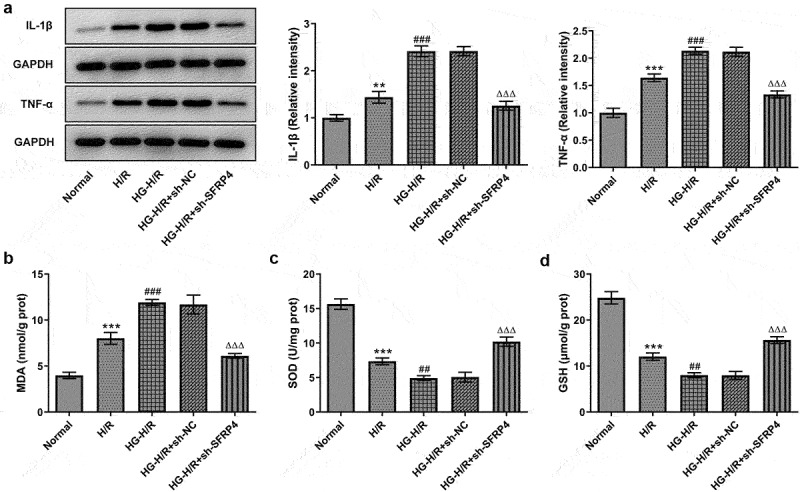
Interference with SFRP4 inhibits inflammation and oxidative stress in high glucose-induced H/R cardiomyocytes cells. (a). Western blot was applied to identify the expression level of inflammatory factors. (b-d). The levels of oxidative stress-related factors MDA, SOD and GSH were determined using the ELSIA kit. Results are the mean ± SD. **P < 0.01, ***P < 0.001 versus Normal. ^##^P < 0.01, ^###^P < 0.001 versus H/R. ^ΔΔΔ^P < 0.001 versus HG-H/R + sh-NC.

To verify the effect of SFRP4 interference on cell damage, the protein levels of inflammatory factors and oxidative stress-related factors were detected by both Western blotting and ELISA kit. Figure 3a set up that there had been a gradual rise in the protein levels of inflammatory factors IL-β and TNF-α in the groups of normal, H/R, as well as H/R + HG but a marked decline in these inflammatory factors in the group of H/R model stimulated with HG after SFPR4 interference. Figure 3b presented that MDA level had a trend identical to that of Figure 3a. Nevertheless, in Figure 3c,d, the levels of SOD and GSH were decreased rapidly in the groups of normal, H/R, and HG+H/R, but remarkably elevated in the group of HG induced H/R model after sh-SFRP4 interference. The findings from these studies uncovered that inflammation and oxidative stress response of H/R cardiomyocytes induced by high glucose were affected by SFPR4 interference.

### SFRP4 interacts with PTPN12 and inhibits PI3K/AKT signaling pathway

**Figure 4.**
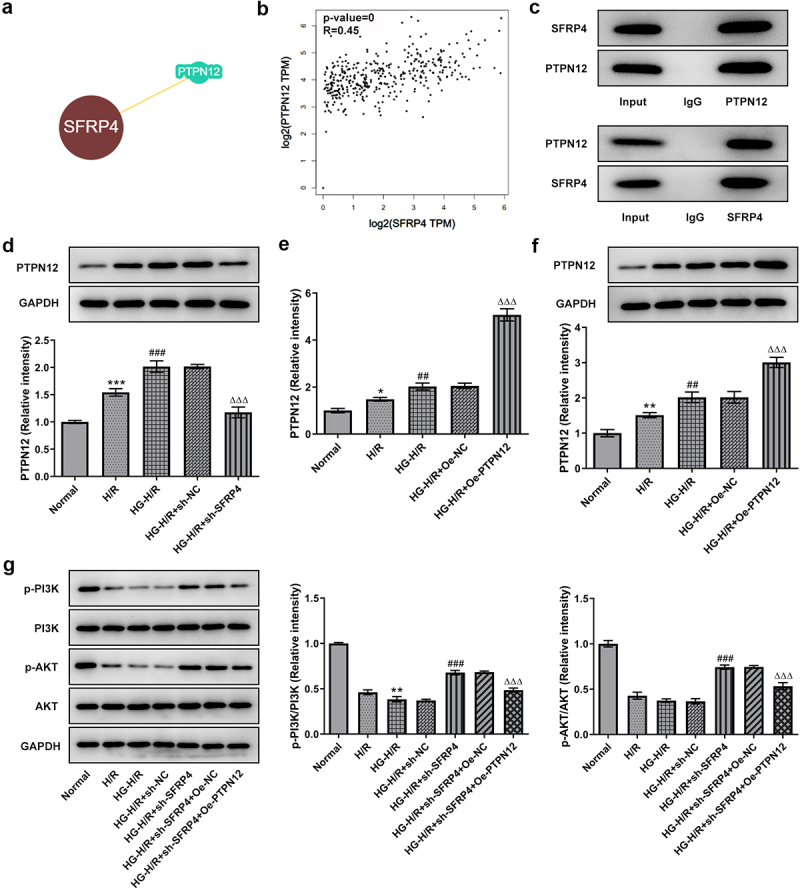
SFRP4 interacts with PTPN12 and inhibits PI3K/AKT signaling pathway. (a). Correlation of SFRP4 with PTPN12 was verified by MINT database. (b). The association between SFRP4 and PTPN12 in the heart was analyzed by GEPIA database. (c). Western blotting assay was used to detect the protein expression level after IP experiment in HG-H/R model. (d). Western blotting was utilized to investigate the expression level of PTPN12. (e). Assessment of PTPN12 overexpression efficiency was conducted by RT-qPCR and Western blotting. (f). Expression levels of PI3K/AKT signaling pathway-related proteins were identified by Western blotting. Results are the mean ± SD. *P < 0.05, **P < 0.01, ***P < 0.001 versus Normal. ^##^P < 0.01, ^###^P < 0.001 versus H/R. ^ΔΔΔ^P < 0.001 versus HG-H/R + sh-NC.

To confirm the correlation between SFPR4 and PTPN12 and their influence on PI3K/ Akt signaling pathway, we demonstrated the relationship between SFPR4 and PTPN12 with the help of two databases MINT and the BioGRID as well as immunoprecipitation assay, and confirmed their effect on P13 K and AKT through the assays of RT-qPCR and Western blotting. As seen from Figure 4a, there was an intimately association between SFPR4 and PTPN12. Additionally, SFRP4 was positively related to PTPN12 in the heart in Figure 4b. It can be easily observed in Figure 4c that SFRP4 directly interacted with PTPN12. Subsequently, we examined PTPN12 expression in HG induced H/R model and found that PTPN12 expression was moved gradually higher, but rapidly declined along with the transfection of sh-SFRP4 (Figure 4d). The transfection of PTPN12 overexpression contributed to the increase in the level of PTPN12 in HG induced H9c2 cells (Figure 4e). Not only that, we also performed the Western blotting assay to examine the protein level of PI3K/AKT signaling pathway-related proteins p-PI3K and p-AKT, which were dramatically declined in H/R model group compared with normal control, and then decreased again in HG induced H/R model. After transfection with sh-SFRP4, protein levels were significant rebound but minor declined when PTPN12 was overexpression in the HG induced H/R model (figure 4f). Taken together, these results revealed that SFRP4 interacted with PTPN12 and inhibited PI3K/AKT signaling pathway.

### PTPN12 overexpression reverses the inhibitory effect of sh-SFRP4 on high-glucose-stimulated H/R cardiomyocyte injury

**Figure 5.**
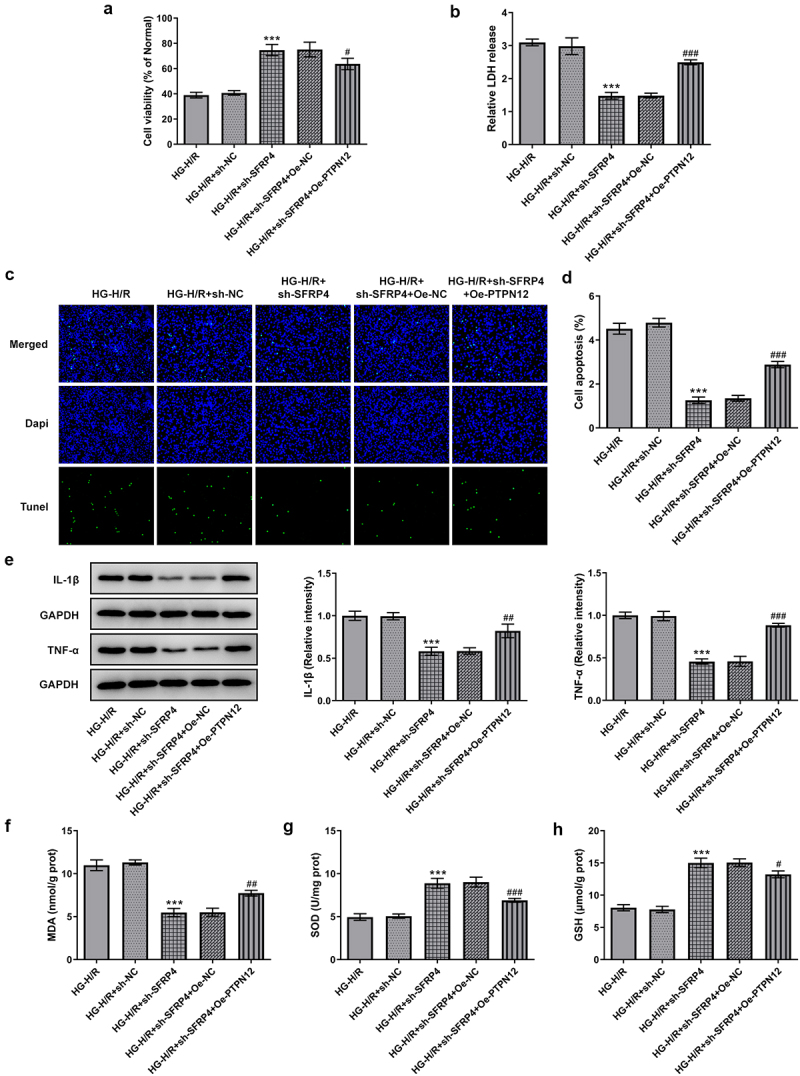
PTPN12 overexpression reverses the inhibitory effect of sh-SFRP4 on high-glucose-stimulated H/R cardiomyocyte injury. (a). CCK-8 assay was in detection of cell viability. (b). LDH kit was used for the detection of LDH release. (c). TUENL (fluorogram) was adopted to examine the level of apoptosis. (d). Expression levels of inflammatory factors were investigated by Western blotting. (e-g). Expression levels of oxidative stress-related factors MDA, SOD and GSH was identified using the ELSIA kit. Results are the mean ± SD. ***P < 0.001 versus HG-H/R + sh-NC. ^#^P < 0.05, ^##^P < 0.01, ^###^P < 0.001 versus HG-H/R + sh-SFRP4 + Oe-NC.

To demonstrate whether PTPN12 overexpression can affect high-glucose-stimulated H/R cardiomyocyte injury, we examined cell viability and expressions of inflammatory factors, oxidative stress factors. Figure 5a shows a decreased cell viability of H9c2 cells after the overexpression of PTPN12 compared with the negative control. Details shown in Figure 5b, PTPN12 overexpression promoted the release of LDH. Moreover, as shown in Figure 5c, PTPN12 overexpression had a certain effect on apoptosis of HG induced H/R model with the transfection of sh-SFRP4. What can be clearly seen in Figure 5d was the elevation of the levels of IL-1β and TNF-α caused by overexpression of PTPN12 and the transfection of sh-SFRP4. Furthermore, we can see in Figures 5e-g that MDA showed an elevated trend similar to that of Figure 5d, while SOD and GSH expressions were declined compared with the negative control. The evidence from this study indicated that PTPN12 overexpression reversed the inhibitory effect of sh-SFRP4 on high-glucose-stimulated H/R cardiomyocyte injury.

## Discussion

Diabetic cardiomyopathy is one of the leading causes of death in diabetic patients. As mentioned previously, SFRP4 may pose potential implications on diabetic myocardial ischemia-reperfusion. The database search revealed that SFRP4 can bind to PTPN12 and was highly positively correlated. And PTPN12 can activate the P13 K/APK signaling pathway. Therefore, we made a speculation that downregulation of SFRP4 inhibits H/R injury in diabetic cardiomyocytes through activation of PI3K/AKT signaling pathway by PTPN12. To simulate the diabetic environment, cells were stimulated with high glucose at a concentration of 30 mmol/L for 24 h. In addition, to establish an H/R cell model, these cells were cultured in a serum-free medium overnight and were subjected to an incubator with a mixture of 95% N2, 5% CO2 and 1% O2 at 37°C for 12 h to simulate anoxia. Finally, cells were exposed in a normoxic chamber for 12 h reoxygenation. In the present study, the experimental data showed that SFRP4 expression was elevated in the H/R model. SFRP4 interference promoted the cell viability of high glucose-stimulated H/R cardiomyocytes and inhibited inflammatory and oxidative stress responses. In addition, SFRP4 could interacte with PTPN12 to inhibit the P13/APK signaling pathway. However, PTPN12 overexpression reversed the inhibitory effect of sh-SFRP4 on high-glucose-stimulated H/R cardiomyocyte injury.

SFRP4 is the largest member of the SFRP family [25] and is associated with many diseases, including obesity, cancer and type 2 diabetes (T2D). SFRP4 is useful as a biomarker of T2D in the clinical diagnosis of the disease. As mentioned in the introduction, bioinformatics analysis found that SFRP4 was upregulated in diabetic cardiomyopathy. In our study, we established an in vitro H/R injury model using HG-induced H9c2 cardiomyocytes. According to our study, it was found that SFRP4 expression was upregulated in a high-glucose-stimulated H/R cardiomyocyte model, which was in accordance with the previous description. On the other hand, SFRP4 is normally expressed in most human organs and has been reported to have a pro-apoptosis role in many tissues [26]. For example, SFRP4 suppresses glioma stem-like cells through reversing epithelial-to-mesenchymal transition, triggering apoptosis and diminishing cancer stem cell properties [27]. SFRP4 induces epithelial cell apoptosis at the onset of breast degeneration [28]. In this study, we constructed the SFRP4 interference plasmid. The experimental results showed that interfering with SFRP4 promoted the viability of high glucose-stimulated H/R cardiomyocytes. In addition, SFRP4 can regulate the Wnt/ Ca2 + signal pathway [29] which mediates the inflammatory response [30]. An increase in the pro-inflammatory factor TNF-α, IL-6 and IL-1β can lead to inflammation. Also, an increase in oxidative stress provokes the production of various inflammatory mediators, including TNF-α, IL-6 and IL-1β [31]. Among them, IL-1β mediates overexpression of SFRP4 by reducing Ca2 + channel expression in pancreatic islet cells, leading to inhibition of insulin-containing granules extravasation [32]. Our experimental results showed that pro-inflammatory factors IL-1β, TNF-α were elevated in the H/R cardiomyocyte model under high glucose induction, but significantly reduced by SFRP4 interference. A similar trend was observed for the expression of oxidative stress-related factor MDA, but the opposite trend was noticed for the expression of SOD and GSH. All of this suggested that SFRP4 interference inhibited the inflammatory and oxidative stress in high glucose-induced H/R cardiomyocytes.

Numerous articles related to PTPN12 have focused on its intrinsic association with tumors. PTPN12 is a tumor suppressor gene that is involves in tumorigenesis and development [33]. For instance, PTPN12 can effectively inhibit the tumorigenicity and metastasis of breast cancer cells [34]. Luo et al. noted that reduced PTPN12 expression is closely associated with recurrence of hepatocellular carcinoma [35]. Reduced PTPN12 expression may serve an important part in increased aggressiveness of nasopharyngeal carcinoma [36]. As mentioned earlier, silencing PTPN12 activates the PI3K/ Akt signaling pathway. Many studies have shown that activation of PI3K/ Akt can reduce I/ R-induced damage. For instance, TSLP protects against hepatic I/R injury by activating PI3K/Akt pathway [37]. VINP protects against brain I/R injury by activating the PI3K/ Akt pathway to phosphorylate CX43 [38]. In addition, activation of PI3K/AKT also attenuates diabetic cardiomyopathy injury. A case in point is that Cavalol alleviates diabetic cardiomyopathy by regulating the PI3K/ Akt /GLUT4 pathway in diabetic mice [39]. According to the database, SFRP4 can bind to PTPN12 and there is a positive correlation between them, which was confirmed in our study by immunoprecipitation experiment. We subsequently examined the expression of PTPN12 and found that it was increased in the H/R cardiomyocyte model under high glucose induction but was suppressed by interference with SFRP4. Next, we constructed an overexpression plasmid of PTPN12 and found that PTPN12 expression was increased rapidly. However, the expression of P13 K/AKT-related proteins was decreased, indicating that the upregulation of PTPN12 can suppress the level of P13 K/AKT. Cell viability, expressions of inflammatory factors and oxidative stress-related factors were reduced in the H/R cardiomyocyte model under conditions of SFRP4 interference and PTPN12 overexpression, which also confirmed that PTPN12 overexpression reversed the inhibitory effect of sh-SFRP4 on high-glucose-stimulated H/R cardiomyocyte injury. Although we observed changing expression levels of p-Akt and p-PI3K, it is not necessary to directly connect these molecules to the SFRP4/PTPN12 axis. It is necessary to perform additional experiments to prove that SFRN4/PTPN12 axis directly connects with Akt and PI3K. Moreover, SFRP4 is engaged in glucose and lipid metabolism by interacting with Wnt ligands [40]. SFRP4 is released from WATs in the period of obesity, leading to elevated production of adiponectin [29]. Since adiponectin may be a factor that influences the results of our experiments, this factor was not considered in the establishment of the H/R cardiomyocyte model in this study. Therefore, identifying the role of SFRP4 in obesity and its molecular mechanism of how it leads to obesity and eventually to diabetes may help to design new therapeutic approaches to treat obese patients suffering from diabetes.

## Conclusion

Overall, our study elucidated that downregulation of SFRP4 attenuated cardiomyocyte injury in H/R models and regulated the activation of the P13 K/AKT signaling pathway through PTPN12, which possibly provides a novel therapeutic target for patients with diabetic cardiomyopathy.

## Supplementary Material

Supplemental Material
