# Supplementary material for: Downregulation of secreted frizzled-related protein 4 inhibits hypoxia/reoxygenation injury in diabetic cardiomyocytes by protein tyrosine phosphatase nonreceptor type 12
Source: Bioengineered. 2022 Mar 15;13(3):7697–708. doi: 10.1080/21655979.2022.2034706 (PMC9278962; doi:10.1080/21655979.2022.2034706)
Supplement: Supplemental Material [file KBIE_A_2034706_SM1689.docx]

Fig1B

SFRP4





GAPDH





Fig2B

SFRP4





GAPDH





Fig 3A

IL-1β





GAPDH





TNF-α





GAPDH





Fig 4C

SFRP4





PTPN12





SFRP4





PTPN12





Fig 4D

PTPN12





GAPDH





Fig 4F

PTPN12





GAPDH





Fig 4G

p-PI3K





PI3K





p-AKT





AKT





GAPDH





Fig 5E

IL-1β





GAPDH





TNF-α





GAPDH
